# Systemic Lupus Erythematosus: Pathogenesis at the Functional Limit of Redox Homeostasis

**DOI:** 10.1155/2019/1651724

**Published:** 2019-11-26

**Authors:** Jay Pravda

**Affiliations:** Therashock LLC, 4371 Northlake Blvd #247, Palm Beach Gardens Fl 33410, USA

## Abstract

Systemic lupus erythematosus (SLE) is a disease characterized by the production of autoreactive antibodies and cytokines, which are thought to have a major role in disease activity and progression. Immune system exposure to excessive amounts of autoantigens that are not efficiently removed is reported to play a significant role in the generation of autoantibodies and the pathogenesis of SLE. While several mechanisms of cell death-based autoantigenic exposure and compromised autoantigen removal have been described in relation to disease onset, a significant association with the development of SLE can be attributed to increased apoptosis and impaired phagocytosis of apoptotic cells. Both apoptosis and impaired phagocytosis can be caused by hydrogen peroxide whose cellular production is enhanced by exposure to endogenous hormones or environmental chemicals, which have been implicated in the pathogenesis of SLE. Hydrogen peroxide can cause lymphocyte apoptosis and glutathione depletion, both of which are associated with the severity of SLE. The cellular accumulation of hydrogen peroxide is facilitated by the myriad of stimuli causing increased cellular bioenergetic activity that enhances metabolic production of this toxic oxidizing agent such as emotional stress and infection, which are recognized SLE exacerbating factors. When combined with impaired cellular hydrogen peroxide removal caused by xenobiotics and genetically compromised hydrogen peroxide elimination due to enzymatic polymorphic variation, a mechanism for cellular accumulation of hydrogen peroxide emerges, leading to hydrogen peroxide-induced apoptosis and impaired phagocytosis, enhanced autoantigen exposure, formation of autoantibodies, and development of SLE.

## 1. Introduction

Systemic lupus erythematosus (SLE) is an immune-mediated disease whose originating pathogenesis results in autoantigen exposure giving rise to numerous autoreactive antibodies of varying antigenic specificities that along with a myriad of cytokines are thought to be effectors of disease activity. Genetic susceptibility and environmental factors play important roles in disease development [1, 2]. Studies have shown that repeated immunization in mice not prone to autoimmune disease reproducibly led to the development of systemic autoimmunity [3, 4]. A similar autoimmune response is seen in mice not prone to autoimmunity when macrophages are chemically depleted [5]. Macrophages are required to remove apoptotic cells and prevent autoantigen exposure from apoptotic cells undergoing secondary necrosis. This suggests that increased autoantigenic exposure via increased autoantigenic load or decreased removal is an early event in the pathogenesis of SLE. Macrophages are reported to undergo increased apoptosis when presented with excess apoptotic loads, which increases exposure and decreases removal of autoantigens [6]. When viewed in light of an SLE monozygotic concordance rate as low as 24%, we can reasonably speculate that an important role for environmental factors in the pathogenesis of SLE is to facilitate autoantigenic exposure to the adaptive immune system [7]. This suggests that increased autoantigenic exposure and decreased autoantigen removal are early concomitants in the pathogenesis of SLE.

Several different mechanisms of cell death have been described with the potential of exposing intracellular autoantigens to the immune system [8–10]. However, apoptosis is believed to play a significant role in pathological autoantigen presentation because of the sheer volume of cellular mass normally undergoing apoptosis amounting to 150 billion cells a day or over 10% of total cellular body mass per month [11]. Cells undergoing apoptosis are normally phagocytosed by professional phagocytes such as macrophages; however, studies in individuals with SLE report increased numbers of cells undergoing apoptosis accompanied by impaired phagocytosis [8, 12]. A contemporaneous occurrence of enhanced apoptosis and impaired phagocytosis is considered a key process in the pathogenesis of SLE and can lead to the cumulative exposure of autoantigens resulting in autoantibody production and autoimmunity [8, 13]. This suggests a systemic agent capable of enhancing apoptosis while simultaneously compromising phagocytosis.

Enhanced apoptosis has been associated with depleted glutathione in lymphocytes of patients with SLE [14]. Because glutathione is the major reducing agent responsible for the neutralization of cellular hydrogen peroxide (H_2_O_2_), a reduction in cellular glutathione will result in elevated cellular H_2_O_2_. Hydrogen peroxide is a potent apoptosis-inducing agent [15–19], and studies have demonstrated apoptosis in human lymphocytes exposed to H_2_O_2_ concentrations as low as 0.7 *μ*M, making lymphocytes one of the most sensitive cells in the body to the apoptotic effect of H_2_O_2_ [20]. Hydrogen peroxide is also capable of causing impaired macrophage phagocytosis [21]. When both effects of H_2_O_2_ are combined, a role for H_2_O_2_ in the pathogenesis of SLE is a reasonable consideration. Consistent with this interpretation are studies showing significantly increased serum H_2_O_2_ of up to 220 *μ*M associated with anti-dsDNA antibodies and tissue damage in a murine model of SLE [22]. Contemporaneous lymphocyte apoptosis and impaired macrophage phagocytosis are reported to show a significant correlation with disease activity in individuals with SLE lending further support for a role for H_2_O_2_ in the pathogenesis of SLE [23] Apoptosis and impaired phagocytosis occur once lymphocytes and macrophages have surpassed their functional limit of redox homeostasis allowing the intracellular accumulation of toxic levels of H_2_O_2_. Redox homeostasis refers to a stable equilibrium that arises between the generation of toxic reactive oxygen species and their continuous removal by the cell [24]. Impaired redox homeostasis leads to cellular buildup of H_2_O_2_. The majority of the biological effects of reactive oxidant species are mediated by H_2_O_2_ [25].

**Figure 1:**
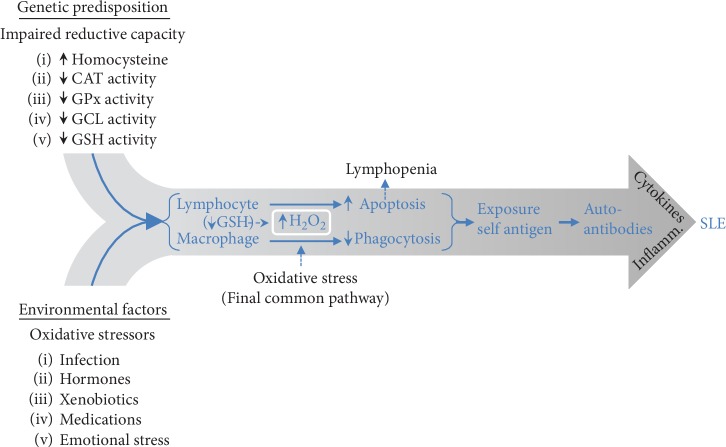
SLE pathogenesis: genetic predisposition stemming from decreased reductive capacity (low glutathione) and diminished reductive reserve due to reduced enzymatic activity in glutathione synthesis combined with environmental factors acting as hydrogen peroxide-generating oxidative stressors results in glutathione (GSH) depletion and increases lymphocyte and macrophage intracellular hydrogen peroxide (a potent apoptotic agent) leading to lymphocyte and macrophage apoptosis. Rising hydrogen peroxide (H_2_O_2_) will impair macrophage phagocytosis before causing apoptosis. Enhanced apoptosis and impaired phagocytosis expose autoantigens to the adaptive immune system that responds with autoantibodies and cytokines resulting in autoimmunity and SLE. Elevated homocysteine (reported in SLE) inhibits glutathione peroxidase (GPX) needed for H_2_O_2_ neutralization [26, 27]. Decreased activity of enzymes needed for H_2_O_2_ elimination such as catalase (CAT), glutathione peroxide (GPx), and glutathione cysteine ligase (GCL) has also been reported in SLE [14, 28–30]. A genetically determined variation of up to one order of magnitude in plasma GSH concentration places a subset of individuals at the lower range of normal and at greater risk of H_2_O_2_-induced oxidative stress [31–33]. Lymphocyte apoptosis contributes to lymphopenia observed in SLE.

Taken together, this suggests that H_2_O_2_ has a causal role in the impaired macrophage phagocytosis in addition to macrophage and lymphocyte apoptosis that leads to the development of SLE. When integrating important disease-modulating elements contained within the exposome and genotype, we can postulate that exposure to environmental oxidative stressors (which generate H_2_O_2_) in a setting of genetically reduced ability to remove H_2_O_2_ contributes to impaired redox homeostasis and elevated cellular H_2_O_2_ facilitating apoptosis, enhanced autoantigenic exposure, autosensitization, and development of SLE (Figure 1). The remainder of this paper will expand upon the causal role of hydrogen peroxide-mediated apoptosis and impaired phagocytosis in response to endogenous and exogenous oxidative stress exposure in the pathogenesis of SLE.

## 2. A Causal Role for H_2_O_2_-Induced Apoptosis in the Pathogenesis of SLE

Hydrogen peroxide has a central role in controlling apoptosis. H_2_O_2_ can initiate apoptosis via caspase-dependent (ASK-1) and caspase-independent pathways. Caspase-independent apoptosis involves H_2_O_2_-induced release of mitochondrial AIF (apoptosis-inducing factor), which directly initiates DNA condensation and apoptosis after translocation to the nucleus. Protein components of the *mitochondrial* permeability transition pore (MPTP) such as the voltage-dependent anion channel in the outer mitochondrial membrane, adenine nucleotide translocator in the inner mitochondrial membrane, and cyclophilin-D in the mitochondrial matrix are targets of H_2_O_2_ and undergo oxidative modifications that will stimulate MPTP opening and apoptosis [18, 34]. H_2_O_2_ is thus a potent multipathway initiator of apoptosis that can trigger mass lymphocyte apoptosis during clonal expansion if cell levels of H_2_O_2_ are allowed to increase.

H_2_O_2_ is continuously generated as a byproduct of cellular metabolic activity including protein synthesis (disulfide bond formation), DNA recycling (xanthine oxidase), fatty acid oxidation (peroxisomal metabolism), and dozens of human enzymes [35–39]. The principal source of cellular hydrogen peroxide is mitochondrial electron transport chain autooxidation during oxidative phosphorylation [38]. Hydrogen peroxide, a potent oxidizing agent, must be neutralized within the cell to prevent toxic accumulation. This is largely accomplished by glutathione-based reductive enzyme systems [40–43]. However, if the production of H_2_O_2_ during a hypermetabolic response overwhelms the cell's reductive capacity, then excess H_2_O_2_ can accumulate within the cell and trigger apoptosis.

In this regard, lymphocyte clonal expansion has been described as a “metabolic bomb” that explodes in a proliferative chain reaction during which glycolysis, the Krebs cycle, and oxidative phosphorylation (electron transport chain) are upregulated to provide the necessary energy in the form of ATP in order to fuel increased metabolic demands that occur during a response to infection [44]. Accompanying this increased metabolic activity is greatly enhanced generation of mitochondrial hydrogen peroxide (H_2_O_2_) as a byproduct of electron transport chain activity and enzymatic reactions involved in the Krebs cycle (alpha-ketoglutamate dehydrogenase) [45, 46]. If H_2_O_2_ generated during cellular activation and highly metabolic states overwhelm the cell's reductive (antioxidant) capacity, the H_2_O_2_ buildup can trigger apoptosis.

Physiological lymphocyte apoptosis occurs during immune catabasis (downregulation after an immune response) after days of clonal expansion, but the appearance of lymphocytopenia during active SLE suggests that this mechanism has been improperly triggered during the initial phases of lymphocyte clonal expansion [47–51]. This is consistent with H_2_O_2_-induced lymphocyte apoptosis and supported by studies showing depleted glutathione and lymphocyte apoptosis during active SLE [52–54]. In addition to enhanced H_2_O_2_ production, a preexisting impaired cellular reductive capacity compromising the cell's ability to neutralize H_2_O_2_ is suggested by studies reporting significantly increased sensitivity of SLE lymphocytes to the cytotoxic effects of H_2_O_2_ compared to control lymphocytes [55]. This is consistent with a preexisting impairment in reductive capacity/reserve and a role for H_2_O_2_ in the pathogenesis of SLE. Thus, a hypermetabolic lymphocyte response can generate excess H_2_O_2_ leading to apoptosis.

Estrogen and emotional stress are two examples of H_2_O_2_-generating hypermetabolic triggers that are associated with SLE as explained below.

### 2.1. Estrogen

Estrogen-induced apoptosis has previously been reported and is dependent upon the estrogen receptor [56]. Lymphocytes express estrogen receptors that initiate signal transduction and enhance lymphocyte metabolism, which generates increased H_2_O_2_ [57, 58]. Additional studies have reported significantly decreased ability to metabolize hydrogen peroxide with a parallel decrease in cellular glutathione when human cell lines were treated with estrogen [59]. A factor contributing to the decrease in glutathione and inability to metabolize H_2_O_2_ during estrogen treatment may be due to the requirement for NADPH in the cytochrome P450 oxidase-mediated catabolism of estrogen [60]. In addition to being consumed in estrogen catabolism, NADPH is also required as a source of reducing equivalents for cellular regeneration of glutathione by glutathione disulfide reductase (EC 1.8.1.7), an important source of cellular glutathione [61]. Thus, estrogen catabolism competes for this critical source of reducing equivalents needed for glutathione regeneration in the cell resulting in sequestration of NADPH away from reduced glutathione regeneration. This can lead to decreased cellular glutathione and the inability to metabolize H_2_O_2_, as observed. This suggests that estrogen's initiating effect in SLE pathogenesis and exacerbating effect in disease progression stem from enhanced metabolic generation of H_2_O_2_ in a setting of genetic and/or induced impaired reductive capacity that can lead to higher levels of lymphocyte H_2_O_2_ with subsequent H_2_O_2_-induced apoptosis and autoantibody formation. This effect would be more pronounced in females as they have higher circulating levels of estrogenic hormones than males.

Hydrogen peroxide-induced apoptosis and release of autoantigens are consistent with studies showing that estrogen enhances severity and flares of SLE in both human and animal models [57, 58]. Finally, since oxidative stress can be additive, the higher levels of estrogenic hormones in females can also lead to a priming effect, increasing sensitivity to apoptosis from exposure to natural and synthetic compounds with estrogenic activity in the food, soil, air, and water that enter the body via the oral, inhaled, and dermal routes resulting in impaired redox homeostasis [62, 63].

“Full-blown” SLE developing in previously healthy women after ovulation induction therapy has been reported [64]. Ovulation induction therapy significantly raises estrogen levels suggesting a physiological effect of estrogen in the pathogenesis of SLE. This is consistent with estrogen-induced hypermetabolic generation of H_2_O_2_ and subsequent H_2_O_2_-induced mass lymphocyte apoptosis.

### 2.2. Emotional Stress

Emotional stress is a recognized factor associated with SLE onset and exacerbation [65, 66]. Stress increases adrenergic hormone secretion from adrenal glands and the sympathetic nervous system [67, 68]. All lymphocytes express adrenergic receptors that are activated by adrenergic hormones [69, 70]. Adrenergic receptor activation dramatically alters lymphocyte proliferation, differentiation, protein synthesis, and cytokine/antibody production and secretion [71, 72]. These large functional changes are reflected in mitochondrial metabolism, and studies have shown that norepinephrine-stimulated lymphocytes generate increased mitochondrial superoxide [73]. Superoxide undergoes immediate enzymatic conversion to H_2_O_2_ at the site of production within mitochondria by the enzyme superoxide dismutase (EC 1.15.1.1) [74]. Lymphocyte activation also causes mitochondrial hyperpolarization leading to a more reduced electron transport chain [75]. This increases electron leakage from the ETC leading to enhanced H_2_O_2_ production.

Thus, acute stressful stimuli can flood the body with adrenergic hormones and in a setting of inadequate reductive capacity/reserve (i.e., decreased glutathione) can lead to accumulation of excess H_2_O_2_ within lymphocytes resulting in mass apoptosis and subsequent intracellular antigen exposure. By this fashion, continued stress-induced autoantigen exposure may contribute to the onset or exacerbation of SLE. Consistent with this interpretation are studies showing that psychological stress increases human lymphocyte apoptosis [76].

### 2.3. Mercury

Mercury is an example of a highly toxic oxidative stressor that can accumulate in the body. Mercury is a ubiquitous contaminant in the environment, and studies have documented significantly elevated blood levels of mercury in patients with SLE that independently correlated with disease activity [77, 78]. Mercury is a reductive depleting agent that irreversibly binds reduced thiol groups present in glutathione and other proteins [79, 80]. This inactivates glutathione, which can no longer fulfil its role in the removal of hydrogen peroxide. Mercury also inactivates glutathione peroxidase, the principal enzyme involved in H_2_O_2_ elimination [81]. The end result facilitates a rise in cellular H_2_O_2_ which can lead to apoptosis. This suggests that chronic mercury exposure is an oxidative stressor that contributes to apoptosis and autoantigen exposure with subsequent autoantibody formation, which may increase the risk of SLE development in the future.

Consistent with this interpretation are studies showing subclinical autoimmunity (anti-DNA antibodies) in reproductive-age human females with low levels of blood mercury generally considered safe [82]. The observed low-level mercury exposure associated with subclinical autoimmunity suggests a contribution from other oxidative stressors. This is supported by murine models of SLE that were worsened by nontoxic amounts of mercury exposure [83]. Thus, mercury exposure can deplete glutathione causing H_2_O_2_ levels to increase, which increases the risk of apoptosis, autoantigenic exposure, and development and/or worsening of SLE.

## 3. Endogenous Oxidative Stressors

### 3.1. Homocysteine

Endogenous metabolites can also contribute to oxidative stress-induced lymphocyte apoptosis and autoantigenic exposure. Homocysteine is a nonproteogenic amino acid breakdown product of protein metabolism whose serum concentration is frequently elevated in children and adults with SLE [84, 85]. Homocysteine has been reported to inhibit GPx activity by 10-fold, and inhibition of GPx was shown to occur at physiologic (9 *μ*mol/L) concentrations of free homocysteine [24, 25]. Homocysteine also downregulates cellular glutathione peroxidase by decreasing translation of this enzyme [86]. As mentioned above, glutathione peroxidase is the principal antioxidant enzyme utilizing glutathione as reductive cofactor for the reduction (neutralization) of cellular hydrogen peroxide. A reduction in the activity of this critical enzyme can increase cellular hydrogen peroxide, which can contribute to lymphocyte oxidative stress, apoptosis, autoantigen exposure, and worsening of SLE. Consistent with this interpretation are studies showing that increased homocysteine serum levels correlate with disease severity in patients with lupus erythematosus [87].

### 3.2. Mitochondrial Heteroplasmy and SLE

Mitochondrial heteroplasmy (MH) is a form of acquired endogenous oxidative stress that is self-amplifying, internally reinforcing, and mutagenic. MH occurs when H_2_O_2_ reacts with mitochondrial DNA (mtDNA) inflicting oxidative damage. This introduces mtDNA mutations, which increase lymphocyte H_2_O_2_ in a self-amplifying vicious cycle that increased cellular steady-state H_2_O_2_ levels that facilitate lymphocyte apoptosis and autoantigenic exposure. The self-perpetuating nature of MH can result in significant, continuous, and mounting endogenous lymphocyte H_2_O_2_. In support of this interpretation and a role for H_2_O_2_ in the pathogenesis of SLE, studies have shown significant mitochondrial heteroplasmy and H_2_O_2_ production in lymphocytes of individuals with SLE, and mitochondrial heteroplasmy is reported to be related to the development and progression of SLE [88–90].

Mitochondrial DNA is highly vulnerable to H_2_O_2_-induced oxidative damage due to the proximity of mtDNA to the electron transport chain (ETC), both of which reside on the matrix side of the inner mitochondrial membrane. Mutated mtDNA will result in base mutations and nucleotide mispairing that, upon transcription, lead to the incorporation of mutated protein subunits into the ETC [91–94]. Mutated ETC components will interfere with electron transport leading to further electron leakage and increased H_2_O_2_ production [95–98]. This establishes a self-amplifying vicious cycle in which H_2_O_2_-induced mtDNA damage results in greater amounts of ETC-generated H_2_O_2_ which, in turn, further damage mtDNA leading to increasingly greater degrees of mitochondrial heteroplasmy and ETC dysfunction with higher steady-state levels of intracellular H_2_O_2_ [93–96, 99, 100]. Once mtDNA oxidative damage (heteroplasmy) has occurred, the entire process is self-perpetuating and self-amplifying. The end result is exhausted glutathione, mitochondrial hyperpolarization, and depleted ATP due to rising mitochondrial generation of H_2_O_2_. Elevated lymphocyte H_2_O_2_ promotes apoptosis and autoantigenic exposure.

Taken together, this suggests a natural history of disease in which cumulative and additive oxidative stress from the interaction of genetic predisposition (decreased reductive capacity/reserve), endogenous sources (hormones, metabolites, and mitochondrial heteroplasmy), and exogenous chemicals (xenobiotics) combines to persistently increase intracellular H_2_O_2_ resulting in enhanced lymphocyte apoptosis. Elevated lymphocyte H_2_O_2_ can cause spontaneous apoptosis or lower the threshold for oxidative stress to trigger apoptosis. This can result in exposure of intracellular autoantigenic material to the adaptive immune system and subsequent development of autoimmunity if apoptotic cells are not efficiently removed by phagocytosis.

However, as described in the next section, impaired phagocytosis is present in SLE suggesting enhanced apoptosis and impaired phagocytosis are core contributory components in the pathogenesis of SLE.

## 4. Immune Shielding: Phagocytosis of Apoptotic Cells

Phagocytosis of apoptotic cells “shields” the immune system from autoantigens. However, H_2_O_2_ can cause impaired phagocytosis in addition to apoptosis of phagocytes (i.e., macrophages) that are needed to remove apoptotic cells. This contributes to autoantigenic exposure as discussed below.

Up to 150 billion cells die every day in the human body [11]. This represents over 10% of total cellular body mass each month. This physiological cell death, known as apoptosis, is programmed to occur as old cells are replaced with new ones. If apoptosis of this large dying cell mass is allowed to proceed unchecked, it would continuously expose intracellular autoantigens to the adaptive immune system as dying apoptotic cells undergo secondary necrosis and intracellular autoantigenic contents are released into the extracellular environment or bloodstream where they would elicit an immune response [101, 102]. This does not normally happen because the adaptive immune system is mostly shielded from exposure to autoantigens by phagocytes (i.e., macrophages) that can identify cells undergoing apoptosis and target them for phagocytosis, which safely degrades autoantigens, preventing immune activation [10, 13, 103, 104].

However, phagocytosis is impaired in SLE [105]. Impaired macrophage phagocytosis increases autoantigenic load and is additive to enhanced lymphocyte apoptosis because both anomalies increase autoantigenic exposure to the adaptive immune system resulting in autoimmunity and hypercytokinemia. The contemporaneous presence of enhanced apoptosis and impaired phagocytosis suggests a common mechanism leading to both abnormalities.

Engulfment of apoptotic cells by professional motile phagocytes (i.e., macrophages, neutrophils) requires migration to the apoptotic body and subsequent phagocytosis, both of which are energy-intensive processes [11, 106, 107]. This generates large amounts of H_2_O_2_ from both the NADPH oxidase complex and electron transport chain autooxidation [108]. Additionally, macrophages make extensive use of the amino acid glutamine as an anaplerotic precursor to replenish Krebs cycle intermediary metabolites [107, 109]. Glutamine is also required for glutathione biosynthesis in order to effectively metabolize cellular hydrogen peroxide. Thus, under conditions of sustained phagocytic activity (during periods of enhanced apoptosis, i.e., SLE) when demand for glutathione to neutralize cellular H_2_O_2_ is high, the availability of glutathione may be limited due to increased anaplerotic metabolism of glutamine within phagocytes. This can result in glutathione depletion and elevated phagocyte H_2_O_2_ levels. This view is supported by studies showing depleted neutrophil glutathione levels in patients with SLE suggesting elevated cellular H_2_O_2_ [110]. Large energetic and anaplerotic requirements make the process of phagocytosis a significant oxidative stressor to professional motile phagocytes such as macrophages and neutrophils, which increased cellular H_2_O_2_.

The finding in SLE patients that glutathione is depleted in other nonphagocytic cell lines such as erythrocytes and lymphocytes in addition to serum and plasma suggests depletion of blood reductive capacity with subsequent elevation in serum H_2_O_2_ levels [111]. Apoptosis occurring throughout the body in other tissues such as the bone marrow, endothelium, skin, kidney, neutrophils, lymphocytes, and macrophages is consistent with systemically elevated serum H_2_O_2_, which is a potent cell membrane-permeable oxidizing agent capable of inducing intrinsic pathway apoptosis in most any tissue or cell line [53, 112–119]. This is supported by multiple abnormalities in bone marrow stem cells such as increased intrinsic pathway apoptosis, dysfunctional mitochondrial signaling pathways, and increased mitochondrial superoxide production consistent with H_2_O_2_-mediated oxidative damage and H_2_O_2_-induced mitochondrial hyperpolarization [120]. Multiple aberrations in activation status and secretory functions of circulating and tissue-infiltrating monocytes and macrophages are consistent with indiscriminant H_2_O_2_-induced oxidative damage [121].

In other words, excess H_2_O_2_ originating from intra- or extracellular sources can diffuse throughout cells and their organelles causing indiscriminate oxidative damage to multiple targets resulting in a myriad of functional abnormalities. Excess cellular H_2_O_2_ results in impaired phagocytosis and apoptosis of phagocytes (macrophages, neutrophils) in addition to apoptosis of virtually any cell line contributing to autoantigenic exposure and development of or worsening of SLE [16].

## 5. Discussion

The list of abnormalities associated with SLE is voluminous and reflective of the various cytokines and numerous autoantibodies that can affect any part of the body. With an originating pathogenesis that is buried deep in the past at the time of diagnosis, the pathophysiology develops unhindered and unrecognized until clinical disease becomes evident. Extensive research into the pathogenesis of SLE has identified over 100 autoantibodies of different antigenic specificity appearing up to a decade prior to disease onset [122, 123].

The recognition that individuals with SLE have increased apoptosis and impaired phagocytosis suggests a pathogenesis mediated principally by increased adaptive immune system exposure to an ever-increasing autoantigenic load. The dual presence of enhanced apoptosis and impaired phagocytosis points to a common underlying mechanism. When paired with the finding of depleted glutathione in individuals with SLE, this suggests the involvement of H_2_O_2_. Hydrogen peroxide is a potent oxidizing and apoptosis-inducing agent that can also impair phagocytosis. Glutathione is needed to neutralize H_2_O_2_, and in the absence of glutathione, H_2_O_2_ levels will increase.

Studies have shown that SLE serum can induce apoptosis in healthy lymphocytes, and this is mediated via the intrinsic (mitochondrial) pathway independent of death receptors and is unaffected by heat inactivation or IgG absorption [124, 125]. Serum from patients with active SLE accelerated apoptosis of macrophages from healthy subjects, and SLE serum is also reported to impair the phagocytic activity of healthy control macrophages, which was restored upon exposure to normal serum [126]. These observations are consistent with the known effects of H_2_O_2_, which is cell membrane permeable and capable of inducing the intrinsic (mitochondrial) apoptosis pathway [17, 19, 127].

Normalization of macrophage phagocytosis by healthy serum can be attributed to normal serum's ability to act as a reductive sink facilitating diffusion of excess intracellular H_2_O_2_ to the extracellular environment and restoration of normal macrophage redox potential by internalizing reducing equivalents (i.e., cysteine) contained in normal serum in order to replenish depleted cellular reductive capacity (i.e., glutathione) [128].

Taken together, the data suggest that a significant contributing mechanism involved in disease initiation is dependent upon H_2_O_2_-induced glutathione depletion followed by H_2_O_2_-mediated apoptosis and impaired phagocytosis resulting in autoantigenic exposure to the adaptive immune system with subsequent autoantibody and cytokine production culminating in SLE.

**Figure 2:**
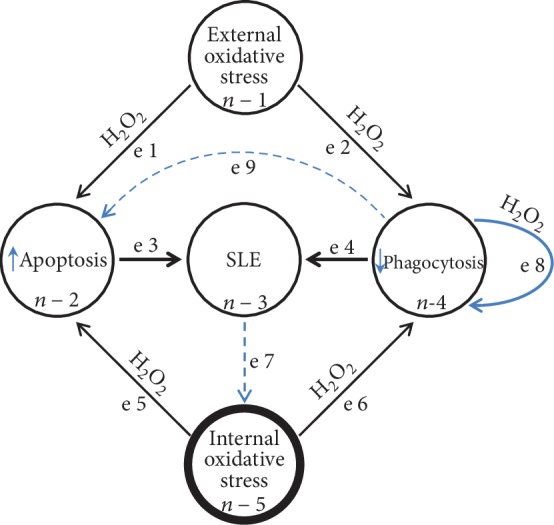
Pathogenesis of SLE: network diagram. Network diagram of SLE showing self-reinforcing positive biofeedback circuits. External (xenobiotics) (n-1) and internal (n-5) oxidative stressors (estrogen, stress hormones, and mitochondrial heteroplasmy) generate lymphocyte and macrophage hydrogen peroxide (H_2_O_2_) (e1, e2, e5, and e6) that increases apoptosis (n-2) and impairs phagocytosis (n-4). The increased autoantigenic exposure leads to cytokine and autoantibody production by the adaptive immune system (e3, e4), which results in autoimmunity and SLE (n-3). Internal oxidative stress is continuous and fuels apoptosis and impaired phagocytosis. The process of phagocytosis itself is an oxidative stressor that can contribute to impaired phagocytosis (e8). Macrophages also undergo H_2_O_2_-induced apoptosis that increases autoantigenic load and reduces phagocytosis (e9). Autoantibodies and cytokines generate H_2_O_2_ due to Fc peroxide activity and signal transduction, respectively (e7), resulting in a self-reinforcing pathological feedback circuit making serological remission a rare occurrence [22, 57, 58, 130–132]. E = edge, n = node.

Oxidative stress, the ability to increase cellular H_2_O_2_, can be caused by multiple environmental chemical xenobiotics and hormones, either by directly depleting glutathione or by signal transduction-induced cellular activation [129]. Thus, continuous endogenous H_2_O_2_-mediated oxidative stress lowers the threshold for disease exacerbation by exogenous oxidative stressors leading to increased lymphocyte apoptosis whose cellular debris is inefficiently removed. Once initiated, a network of pathological positive biofeedback mechanisms leads to a heightened immune reactive state involving multiple immune effector cells including T and B cells (Figure 2). The end result is an alphabet of autoantibodies within a serum cytokine soup replete with H_2_O_2_ that can cause dysfunction of almost any organ in the body making prolonged remission a rarity [130–132].

Lymphocytes are highly sensitive to H_2_O_2_-induced apoptosis. This sensitivity to apoptosis can have a physiological role in order to appropriately downregulate an immune response after infection but can also be co-opted by environmental oxidative stressors that can inappropriately induce apoptosis by increasing cellular H_2_O_2_. Xenobiotic chemicals can remain in the body for extended periods of time causing a cumulative endogenous oxidative stress that contributes to a lower threshold for future oxidative stress-induced disease exacerbations. This suggests that, in most cases, SLE does not start out as a primary immune-mediated disease but evolves into one after reductive capacity is depleted and redox homeostasis becomes impaired resulting in increased apoptosis, impaired phagocytosis, and autoimmunity. This distinction has practical implications for altering the natural history of disease because it suggests that the long latent period between the appearance of autoantibodies and disease onset is the time during which redox homeostasis is becoming progressively more disrupted and when intervention to correct impaired redox homeostasis may prevent development of disease.

## 6. Conclusion

Decades of research attempting to “reverse engineer” systemic lupus erythematosus (SLE) have led to the understanding that autoantigenic exposure triggers immune activation causing symptoms, signs, and organ pathology we recognize as SLE. We are now at the level of identifying an initiating cell type and mechanism whereby this can occur and integrate these elements with compatible genetic predisposition and environmental factors that enhance autoantigenic exposure leading to disease.

The data suggest that H_2_O_2_-induced apoptosis of lymphocytes and macrophages plays a prominent causal role in the pathogenesis of SLE. The high sensitivity of lymphocytes to H_2_O_2_-induced apoptosis at submicromolar exposure levels suggests that this mechanism is a major early initiating event exposing the adaptive immune system to cellular autoantigens. H_2_O_2_ also impairs macrophage phagocytosis of apoptotic cells and additionally triggers macrophage apoptosis. The end effect is enhanced and prolonged autoantigenic exposure as a result of increased apoptosis and decreased phagocytosis of apoptotic cells.

A preexisting genetic disposition which compromises the ability to neutralize H_2_O_2_ in addition to environmental factors that increase lymphocyte H_2_O_2_ (oxidative stress) or decrease cellular reductive capacity contributes to apoptosis and autoantigenic exposure. This suggests that lymphocyte screening for H_2_O_2_ and glutathione levels may predict risk of developing SLE. It also suggests that preventive measures aimed at avoidance of environmental oxidative stressors (all of which increase H_2_O_2_) and augmenting individual reductive capacity (i.e., glutathione) can treat and prevent disease [133, 134]. This implies that fortifying the food supply with reducing equivalents may be able to offer protection on a population level against oxidative stress-induced apoptosis and prevent the development of SLE and other diseases mediated by oxidative stress.
